# Defining Participant Exposure Measures in Web-Based Health Behavior Change Programs

**DOI:** 10.2196/jmir.8.3.e15

**Published:** 2006-08-30

**Authors:** Brian G Danaher, Shawn M Boles, Laura Akers, Judith S Gordon, Herbert H Severson

**Affiliations:** ^1^Oregon Research InstituteEugeneORUSA

**Keywords:** Health behavior, Internet, Web, behavioral research, participant exposure, engagement

## Abstract

**Background:**

Published research on the use of Web-based behavior change programs is growing rapidly. One of the observations characterized as problematic in these studies is that participants often make relatively few website visits and spend only a brief time accessing the program. Properly structured websites permit the unobtrusive measurement of the ways in which participants access (are exposed to) program content. Research on participant exposure to Web-based programs is not merely of interest to technologists, but represents an important opportunity to better understand the broader theme of program engagement and to guide the development of more effective interventions.

**Objectives:**

The current paper seeks to provide working definitions and describe initial patterns of various measures of participant exposure to ChewFree.com, a large randomized controlled trial of a Web-based program for smokeless tobacco cessation.

**Methods:**

We examined measures of participant exposure to either an Enhanced condition Web-based program (interactive, tailored, and rich-media program) or a Basic condition control website (static, text-based material). Specific measures focused on email prompting, participant visits (number, duration, and pattern of use over time), and Web page viewing (number of views, types of pages viewed, and Web forum postings).

**Results:**

Participants in the ChewFree.com Enhanced condition made more visits and spent more time accessing their assigned website than did participants assigned to the Basic condition website. In addition, exposure data demonstrated that Basic condition users thoroughly accessed program content, indicating that the condition provided a meaningful, face-valid control to the Enhanced condition.

**Conclusions:**

We recommend that researchers conducting evaluations of Web-based interventions consider the collection and analysis of exposure measures in the broader context of program engagement in order to assess whether participants obtain sufficient exposure to relevant program content.

## Introduction

One of the common findings of research on Web-based behavior change programs is that participants spend only a relatively meager amount of time accessing their online intervention [1]. This implies minimal participant exposure to the critical behavior change ingredients of the program, which could potentially reduce program impact. In response to this finding, a number of published reports of Web-based interventions have described website usage statistics, including number and duration of visits as well as the number and type of Web pages viewed [2-11]. Research on Web-viewing behavior is rapidly growing in other domains (eg, advertising [12] and technology [13]).

This paper describes participant exposure to a two-arm randomized controlled trial of Web-based programs designed to assist adults in quitting smokeless tobacco (either snuff or chewing tobacco). Following a brief program description, we present a set of unobtrusive measures of website exposure [14] and the results of our exposure analyses. We believe that this level of detail will prove helpful to other researchers investigating the design of optimally effective Web-based behavior change programs.

## Methods

### ChewFree Program for Smokeless Tobacco Cessation

We designed the ChewFree trial to compare the efficacy of two smokeless tobacco cessation websites: Basic and Enhanced.The Basic condition, which represented a subset of the content presented in the Enhanced condition, offered a printable self-help smokeless tobacco cessation booklet, printable cessation resources (eg, describing the use of herbal snuff products, nicotine replacement products), and annotated links to other recommended websites for tobacco cessation. The Enhanced condition offered a tailored and interactive Web-based program that included text-based information (health and behavioral strategies focused on quitting and preventing relapse), video-based testimonials, printable resources, interactive activities, annotated links to other website resources, and two Web forums (a "Talk with Others" social support forum, and an "Ask an Expert" forum for submitting questions to project staff).

ChewFree.com intervention components were based largely on Bandura's Social Cognitive Theory [15-17] in which individuals are viewed as proactive agents who can exercise motivational and self-regulatory skills to change their health habits. According to this theory, individuals choose their environments, seek out beneficial social networks, and engage in other self-management behaviors that allow them to achieve both initial change and long-term maintenance. Multi-component smoking cessation and relapse-prevention interventions have successfully incorporated these strategies [eg, 7,18-20], and adaptations of these same approaches have been found well suited to smokeless tobacco cessation programs [21-23].

**Figure 1:**
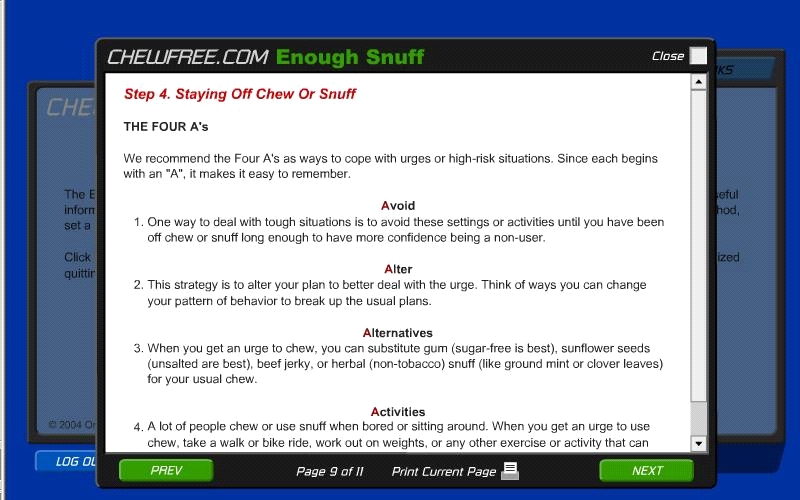
Basic condition (excerpt of Enough Snuff guide)

**Figure 2:**
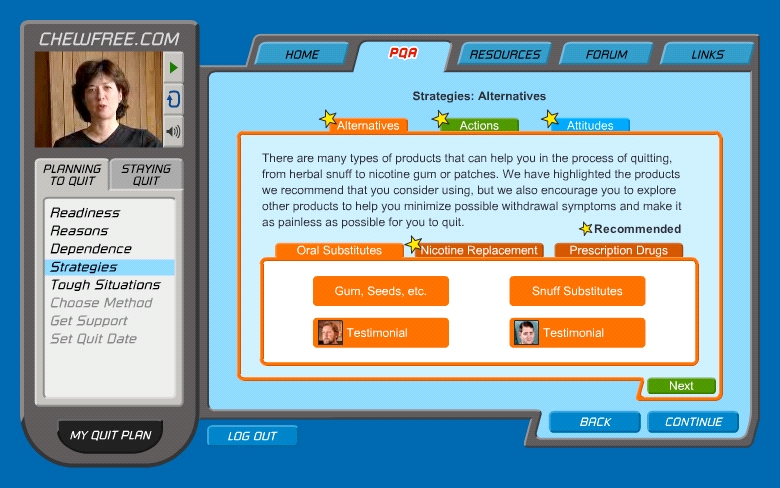
Enhanced condition (excerpt showing video narration by a smokeless tobacco cessation expert to accompany the Personal Quitting Assistant, or PQA)

The Basic and Enhanced Web-based programs offered smokeless tobacco cessation assistance using markedly different information architectures [24]. The Basic condition (Figure 1) presented text-based content using four navigational Web pages: Home, *Enough Snuff*—an adaptation of the smokeless tobacco cessation manual used in prior research [25], Resources, and Links. The Enhanced condition (Figure 2) used five navigational Web pages: Home, Personal Quitting Assistant, Resources, Forum, and Links. The Personal Quitting Assistant used a hybrid information architecture design [24] that guided participants in a step-wise manner through eight modules of the Planning to Quit content while offering optional content along the way. In addition, the information architecture prevented users from accessing content in the Staying Quit module until they returned to the website at a later date and reported that they had either quit using smokeless tobacco or had relapsed. Progress was self-paced in that participants in both the Enhanced and Basic conditions determined when they chose to visit the program and how much content they would cover during each visit.

Content was presented using text, graphics, activities, and two types of videos: a video expert guide who narrated key portions of content that was also presented as text, and video testimonials of smokeless tobacco users whose presentations supported the recommendations of the program. The narration videos were automatically launched (ie, they did not require user selection) for users with high bandwidth connections, but they were not displayed automatically to participants with dial-up access [26]; all users could toggle them on or off as desired.

We designed the Enhanced condition to be attractive by offering a broad spectrum of content tailored to the interests and the smokeless tobacco use/abstinence status of each participant. For example, participants who were preparing to quit were encouraged to review program content focused on Planning to Quit, whereas those participants who indicated that they had quit using smokeless tobacco were encouraged to review content on Staying Quit. In addition, the intervention used multiple methods for delivering content along with engaging activities. Compared to the Basic condition, we predicted that the Enhanced condition would encourage participants to visit more often and for longer periods of time—especially during the first several weeks post-enrollment when attempts to quit and related lapse/relapse experiences would most likely occur.

### Participants

Participants were recruited using a multifaceted marketing campaign that included (1) thematic promotional "releases" to print and broadcast media, (2) Google ads, (3) placement of a link on other websites, (4) limited purchase of paid advertising, (5) direct mailings to smokeless tobacco users, and (6) targeted mailings to health care and tobacco control professionals. This campaign resulted in more than 23500 visits to the ChewFree.com recruitment website from distinct IP addresses over a 1-year period, which yielded 2523 eligible smokeless tobacco users who completed the registration process and enrolled in the ChewFree.com smokeless tobacco cessation research project [27,28]. Participants were randomly assigned either to the Enhanced condition (n = 1260) or the Basic condition (n = 1263).

### Measures of Program Exposure

There is no single universally accepted measure for assessing participant exposure to a Web-based program. Computer-delivered content lends itself well to unobtrusive monitoring of usage patterns. As noted by Peterson [29], there are a number of potentially complementary sources of computer-based monitoring data, such as server log files, cookies, Web beacons, and session identifiers. Many commercial products are available that analyze Web server log files [eg, 30,31]. Cookies offer another powerful tool to tailor website content and monitor usage [32]. Web beacons can be inserted into Web pages to enhance the ability to obtain even more detailed tracking [33]. Because we used user authentication (obtaining username and password at the beginning of every session) with an appropriate scripting language (Macromedia ColdFusion) and SQL database to create the ChewFree websites, we were able to use the session identifier approach to measure exposure [29]. We believe that the session identifier approach offered more flexibility to focus on topics that were relevant to our research than did a commercial log analyzer product focused on issues of commercial importance such as pay-for-click analyses, average revenue per order, top products, and customer segment analysis.

For the present paper, we examined data from participants as of January 10, 2006, which, for most participants, represented approximately 12 months after enrollment (mean = 367.1 days, SD = 116.9; no significant differences between conditions). The minimum number of days since enrollment was 181 days and, in each case, the scheduled date of the 6-month follow-up assessment had elapsed. Textbox 1 summarizes the measures we used, each of which is described below. Detailed measures are provided in the Multimedia Appendix.

**Measures of program exposure**

**Table:** 

**Email prompts**	Percentage of participants sent treatment-related email prompts
**Participant visits**	Number of visits
	Aggregate duration of visits
	Number of daily visits post-enrollment
	Number of days of program access post-enrollment
**Web page views**	Overall number of Web page views
	Specific Web page views (selected smokeless tobacco cessation content)
	Web forum postings

### Treatment-Related Email Prompts

Participants in the Enhanced condition received a variety of email prompts during the study that were not related to assessments. These prompts fell into three categories:

1. Intervention: Participants were sent up to three email messages prior to their quit date, tailored to their chosen method of quitting (cold turkey, nicotine fading, brand switching, blending), and one message on their quit date.

2. Support: We sent three supportive emails timed at fixed intervals after the participant's self-reported quit date.

3. Re-engagement: Participants who failed to log in on a regular basis were typically sent multiple tailored email messages encouraging them to resume accessing the program.

### Participant Visits

Typical measures of visit data include number of visits per participant per condition and both average and total visit duration. We programmed the ChewFree website to record the date/time stamp of the start and end of each participant visit (also referred to as a "session") and for each Web page the participant viewed during each visit. These date/time stamps allowed us to examine both the number of unique visits per participant and session duration.

Because participants were able to abruptly end their use of the program by closing their browser window, there were occasions when we did not capture the date/time data for the end of the session. To analyze these instances, we conservatively approximated the end of the visit by using the date/time of the last Web page that had been accessed before the abrupt end of the session. In addition, we followed the operational definition for visit expiration recommended by Peterson [29]; that is, any Web page viewed for 30 minutes or more was defined as having ended the visit using the ending date/time stamp of the Web page that immediately preceded the hiatus. Moreover, if, after the hiatus, the participant resumed activity, it was considered to be a new visit for measurement purposes.

Participants in both conditions were required to complete an online baseline assessment prior to accessing the program. In addition, all participants received email reminders to complete online follow-up assessments at 6 weeks, 3 months, and 6 months. The email prompt contained a link that caused the log-in page of the Web-based program to appear, followed by presentation of the online assessment. At the end of each assessment, users were returned to their respective website, at which time they were free to explore the website and review its contents. When counting distinct visits that involved program content review, we excluded those visits associated with online assessments unless the participant also explored website content.

### Website Visit Duration

We focused our analysis on aggregate duration (collapsed across visits) because we were concerned with the overall amount of participant exposure to the program. Although we did not choose to do so for purposes of this paper, we could also have examined the changing patterns in the duration of individual visits over time.

### Visits Following Enrollment

We examined the time course for each participant visit by calculating the number of days in which a visit occurred since the date the participant completed the baseline assessment and formally began the study. It is important to note that at the end of the baseline assessment, each participant was automatically presented with the home page of the condition to which he/she was randomly assigned. If, following the end of the assessment, a participant continued to explore the Web-based content, then that event was counted as a unique visit and assigned a value of zero (since zero days had elapsed since the end of the baseline assessment). If a participant had multiple visits on any given day, then this analysis counted each of those visits in the total for that day (ie, participants could have multiple visits per day). We limited our analysis to those visits in which Web-based program content pages were accessed.

In addition to measuring the number of visits per day, we used Kaplan-Meier survival analyses [34-36] to examine the pattern of reduced program participation, also known as *nonusage attrition*[1]. For purposes of this analysis, each participant's last visit that involved review of program content was designated as the date that program usage ended. Duration was defined as the number of days that elapsed since program enrollment (the start of the program) and the date of the last visit. More technically, the population survivor function represents time versus the probability that a randomly selected program participant will continue to access the program. Since all participants stopped using their assigned website in the analysis period (the defined terminal event), no cases were censored. In addition to examining the survival curve, we also report on the estimated median lifetime for each condition, which describes how much time passed before 50% of the sample stops accessing the Web-based program [34].

### Viewing Smokeless Tobacco Cessation Content

Finer grained within-visit analyses focused on participants' viewing of Web pages that presented specific content designed to encourage smokeless tobacco cessation. Because we recorded the date/time of each Web page viewed during each visit, it was possible to calculate the percentage of participants who viewed specific types of Web pages (using the participant sample in each condition as the denominator). For example, we were able to measure the extent to which participants in either condition accessed a ChewFree.com Web page that provided links to other websites offering smokeless tobacco cessation information and assistance (eg, the National Cancer Institute, the National Spit Tobacco Education Program, and the Oral Health America Foundation).

In the Enhanced condition, we also measured participants' use of ChewFree.com Web pages that offered more interactive features, including whether they viewed pages that automatically played video testimonials, whether they accessed a Web page that offered a print feature (and triggered a print dialog box), and whether they listed the names of people whom they believed could offer useful support for smokeless tobacco cessation. And although this paper focuses on exposure rather than on outcome results, we also report on the extent to which participants in the Enhanced condition used the Web page designed to help them choose a quit date for stopping the use of smokeless tobacco.

### Web Forum Data

Finally, we captured data on the extent to which participants in the Enhanced condition used the available peer Web forum ("Talk with Others") or expert forum ("Ask an Expert"). Forum use was logged into the database when participants posted messages, either by creating a new message or responding to an existing message. Unfortunately, we did not track passive viewing of the forum messages, nor did we collect data that would allow us to calculate the amount of time spent viewing forum content.

## Results

### Treatment-Related Email Prompts

Analysis revealed that 63.3% of participants (760/1220) in the Enhanced condition set a quit date and were sent a program-generated series of tailored email prompts associated with preparing to quit. After having been sent at least one of these emails, 10.7% of these participants (81/760) requested to opt out of receiving further emails. A total of 40.7% of participants (488/1220) who reported having quit using smokeless tobacco during the course of the program were eligible to be sent a series of emails supportive of continued abstinence. However, the number of participants who were sent these supportive emails was reduced to 34.8% (425/1220) because 63 had opted out of receiving program-generated emails. Enhanced condition participants who had not exercised the opt-out option (90%; 1079/1200) were also scheduled to receive emails at 7, 30, and 60 days since last log-in, encouraging them to re-engage with the site. We plan to conduct future analyses to assess the relation between the automated email prompts, website usage, and outcome results.

### Number and Duration of Unique Visits

Our initial analysis showed that 0.6% of participants (7/1260) in the Enhanced condition and 0.8% of participants (10/1263) in the Basic condition never visited their assigned website after completing the baseline assessment and becoming enrolled. An additional 3.7% of participants (47/1260) in the Enhanced condition and 5.9% of participants (74/1263) in the Basic condition returned following enrollment but did so only to complete online assessments. These individuals never viewed any Web pages that contained smokeless tobacco cessation content. Removing these participants from our analyses reduced the sample to 2375 participants (1200 in Enhanced condition; 1175 in Basic condition) for whom visit duration could be measured (Table 1).

Rather than being normally distributed, the observed patterns of website visit frequency and duration displayed a significantly positive- or right-skewed distribution, with most cases having occurred at lower values (more frequent and longer visits occurring soon after enrollment). We used the nonparametric Mann-Whitney *U* test to compare these results by condition. Participants in the Enhanced condition made significantly more visits than participants in the Basic condition (*z* = -16.64, *P* < .001, 2-tailed). We also calculated the length of each visit by summing the length of each page view within each visit. Participants in the Enhanced condition spent significantly more time viewing website content collapsed across all Web pages and visits (*z* = -17.63, *P* < .001, 2-tailed).

**Table 1:** Visit details by participant

		**Visits** by Participant^*^		**Overall Visit Duration** by Participant^*^(min)	
**Condition**	**N**	**Median**	**Interquartile** **Range**	**Median**	**Interquartile** **Range**
Enhanced	1200	2.00	3 (1-4)	28.99	37.75 (13.60-51.35)
Basic	1175	1.00	1 (1-2)	12.50	15.83 (6.60-22.43)

^*^P < .001

### Visits Following Enrollment

**Figure 3:**
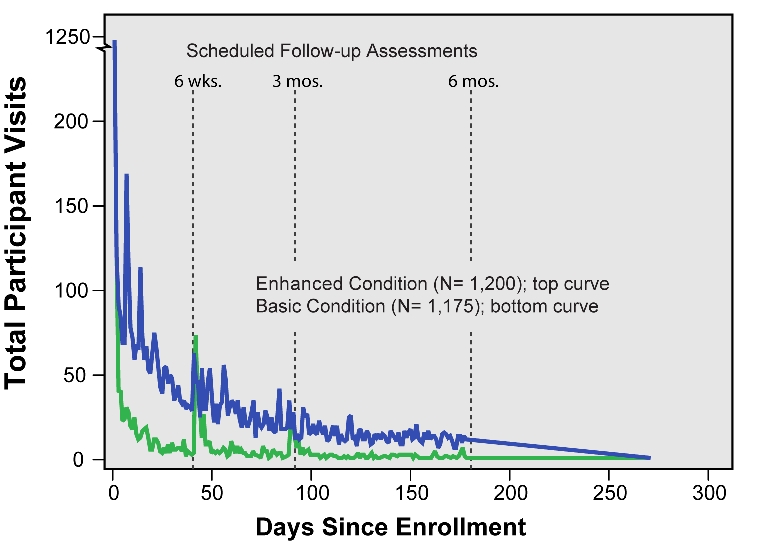
Visits following enrollment

Visits by time course for those 2375 participants who viewed smokeless tobacco cessation content are depicted in Figure 3. Note that if a participant only viewed website content on the day of his/her enrollment, then that individual would be listed in this analysis as having 0 days (zero days since the day of enrollment). In this analysis, a participant could have multiple visits in any given day.

We observed 3783 visits for participants in the Enhanced condition and 2054 visits in the Basic condition. Consistent with usage patterns reported in other research of Web-based interventions [1], participants in our study visited their assigned website more frequently and in greater numbers immediately following enrollment. Thereafter, we observed a steady decrease in visits over time with rapid drops occurring soon after enrollment followed by a slower reduction in visits toward zero asymptote. Even though the analysis did not include all visits that focused only on online follow-up assessments, it nonetheless appears that visits for program content were related to the timing of online assessments and their reminder emails (note vertical markers for the assessments at 6 weeks, 3 months, and 6 months) such that assessment dates appeared to reduce the rate (the steepness of the curve) of declining visits. It is important to note in this regard that upon completion of the online assessment, each participant was returned to the website home page, which would encourage them to review program content.

**Figure 4:**
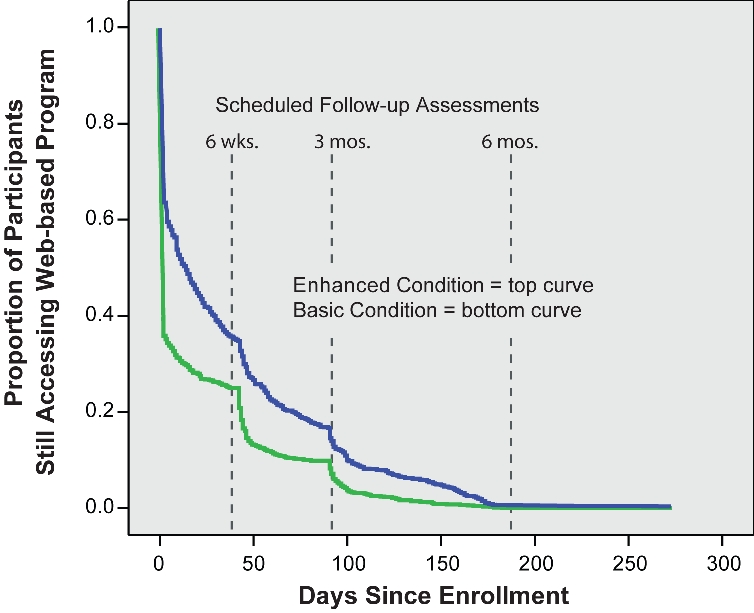
Website activity following enrollment (survival curve)

We also examined the number of days following enrollment that participants continued to access their assigned website for program content (excluding visits to take online assessment only). For purposes of this survival analysis, the last content-accessing visit for each participant was designated as the final date of program usage. For example, 36.1% of participants (433/1200) in the Enhanced condition and 60.7% (713/1175) in the Basic condition stopped using the program on the day they enrolled in the program. Because Figure 4 depicts the percentage of participants who continued to use the program (the "survivors"), it shows that 63.9% of participants in the Enhanced condition and 39.3% of participants in the Basic condition continued to use the program after Day 0 (enrollment day).

We assumed that each participant, regardless of condition, would eventually stop using the Web-based program. Thus we examined the differential pattern of program use atrophy. As depicted in Figure 4, website access essentially stopped by 6 months following program enrollment. The estimated median lifetime website usage (date when 50% of participants stopped using the program) was 11 days for the Enhanced condition and 0 days (ie, the enrollment day) for the Basic condition. A Kaplan-Meier survival analysis indicated that, following enrollment, participants in the Enhanced and Basic conditions exhibited significantly different patterns of continued access to the Web-based program. Both log-rank (Mantel-Cox) and Breslow (generalized Wilcoxon) tests were highly significant (*P* < .001), with the Enhanced condition showing a slower decay (less nonusage attrition) over time than the Basic condition. As noted in the analysis of total visits following enrollment (Figure 3), we observed that reduced program usage was related to the prompting effects of the follow-up assessments at 6 weeks, 3 months, and 6 months.

### Viewing Web Pages With Smokeless Tobacco Cessation Advice

In addition to metrics of overall website exposure, we were interested in the extent to which participants accessed content that contained specific information most relevant to smokeless tobacco cessation and tobacco abstinence. Table 2 displays data on the viewing of selected Web pages that contained information on smokeless tobacco cessation. It is interesting to note that when similar pages were available on both websites (those presenting outside links and the opportunity to print content), a higher percentage of participants in the Basic condition accessed that content than did participants in the Enhanced condition. Similarly, almost 88% of participants in the Basic condition compared with 12.2% of participants in the Enhanced condition viewed every page in an 11-page self-help smokeless tobacco quitting guide. This guide, adapted from the *Enough Snuff* guide [37], was deeply embedded in the Enhanced condition website, thus making it somewhat more difficult to use.

**Table 2:** Web page viewing by participants who accessed at least one Web page containing smokeless tobacco cessation content

**Condition**	**N**	**Outside Links (%)**	**Print Content (%)**	**SmokelessTobaccoQuitting Guide (%)**	**Video Testimonial (%)**	**List of Support People (%)**	**Set Quit Date (%)**	**Staying Quit Content After Quitting (%)**
Enhanced	1200	18.2	78.3	12.2	68.2	24.7	63.3	32.8
Basic	1175	32.1	96.3	87.5				

### Web Forum Usage

We found that 38.2% participants in the Enhanced condition (481/1260) posted content to the Web forum for peers, with 5.2% (65/1260) posting at least one message in the expert forum (Table 3). Each participant who posted a message to the "Ask an Expert" forum also posted at least one message to the peer forum. The distribution of forum postings was right- or positively-skewed, with most cases clustered at lower values (fewer postings). The median number of postings was 2 in the peer forum and 1 in the expert forum. The interquartile range was 11 postings (1-11.50) for the peer forum and 1 posting (1-2) for the expert forum. Using the nonparametric Spearman rank correlation test, we found that forum postings were significantly correlated with visits (*ρ* = .512, n = 481, *P* < .001, 2-tailed) and Web page views (*ρ* = .340, n = 481, *P* < .001, 2-tailed) for participants in the Enhanced condition.

**Table 3:** Web forum activity in the Enhanced condition (n = 1260 users)

	**Users**		**Postings by User**	
**Forum Type**	**n**	**%**	**Median**	**Interquartile** **Range**
Peer	481	38.2	2	11 (1-11.50)
Expert	65	5.2	1	1 (1-2)

## Discussion

It is important to acknowledge several limitations to the present study. First, we did not design the Enhanced intervention website to track passive Web forum viewing. This limitation prevented us from analyzing the duration of Web forum visits by participants who observed postings but did not post their own comment on the forum posts of others. In addition, although study inclusion criteria required all study participants to be able to access their personal email at least once per week, we did not collect data on participants' previous experience using the Internet or on their computer self-efficacy [38]. As a result, we cannot report directly on whether there were significant differences between the intervention and control conditions for these dimensions. We believe that random assignment and our large sample size (N = 2375) would tend to mitigate the likelihood of this effect.

It is thought that a key ingredient in determining the impact of any Web-based behavior change program is the extent to which participants are exposed to the program. This assumption is consistent with the finding that the efficacy and intensity of treatment programs tend to be positively related. For example, research on smoking cessation interventions—including self-help approaches—has illustrated the relationship between abstinence rates and program intensity, typically defined as contact time and number of sessions [4,39,40]. Williams et al [41] have coined the term program *thickness* to refer to the "collective intensity, duration, delivery agent, and intervention modality" of an intervention. However, research has also shown that more is not always better when considering which ingredients to include in an intervention [42] or a website [19,43,44], perhaps because adding features increases the response cost of participation and reduces usage.

Some reviewers of this burgeoning field have recommended that fuller participation in Web-based interventions might be encouraged through the use of a "warm-up period" during which users can demonstrate their commitment by complying with precursor tasks while they become more familiar with what will be asked of them during the course of the program [1,45]. The use of intensive treatment approaches and preliminary litmus tests of commitment must be tempered by recognizing the continuum between clinic-based and public-health models for intervention. Specifically, it may be not be a practical goal to provide a highly intensive, population-wide intervention. Moreover, the use of preliminary barriers may help to reduce attrition in efficacy trials but reduce our ability to conduct effectiveness trials that have a broader reach and greater potential to achieve public health impact [46,47]. A challenging—and fruitful—line of research lies in identifying the proper program ingredients that provide a balance between sufficient exposure to relevant content and structure on the one hand while encouraging widespread user participation and engagement (both recruitment and follow-through).

We found that the estimated median lifetime website usage (date when 50% of participants stopped using the program) was 11 days for the Enhanced condition and 0 days (ie, the enrollment day) for the Basic condition. We anticipate that some measures of exposure and outcome will likely share a curvilinear (inverted U-shaped) relationship such that those individuals who are least ready to make a meaningful change may be more likely to visit the Web-based program for a short time, while participants who are most prepared to change their behavior may similarly choose to visit the Web-based program for a relatively short time. Those participants who are interested in quitting and decide to learn more about how to do so will spend relatively more time visiting the program. It remains for future research to differentiate characteristics that illuminate the pattern of this relationship among motivation, readiness to quit, and program usage.

Measures of participant exposure can help researchers and program developers determine the extent to which content is viewed. These data can point to needed changes in the information architecture and design features of the website. It is reasonable to assume that program content cannot be helpful if it is never viewed. Exposure measures may have utility in that they inform us about whether certain content—or clusters of content—is related to outcome and thus might be considered to be active ingredients in accomplishing the desired behavioral goals. They enable us to better understand idiosyncratic patterns of program use, highlighting ways we can adapt program structure and content to better accommodate (be tailored to) individual differences in participant interests, needs, and learning styles.

In this regard, we intend to examine a variety of relationships between and among measures of exposure and the smokeless tobacco and tobacco cessation outcome measures in the ChewFree.com research project. For example, we will test whether participants who set a quit date are more successful in quitting, as well as whether, after quitting, there is a relationship between accessing content from the Staying Quit module (number and duration of visits) and lasting abstinence. Similarly, we will examine whether those participants who spend more time reviewing program content after they have lapsed are better able to regain control over their behavior and regain abstinence. We also plan to perform content and text analyses of Web forum postings [eg, 48] to explore whether smokeless tobacco cessation might be related to message types, whether cessation and maintenance strategies shared in postings are consistent with program recommendations, and the extent to which postings convey differing levels of confidence and self-efficacy across participants as well as within participants over time.

There is a significant risk of collecting so much detailed exposure and engagement data that the task of analyzing and interpreting results becomes difficult. We suggest that this task can become more manageable and, thus, more fruitful, by focusing its scope through the use of a rationale that incorporates both theory and pragmatism. Potentially relevant rationales are not difficult to identify. Consider, for example, a rationale that builds on the Web foraging model [49,50], which posits that Web users guide their review of online content by quickly identifying interesting *information scents* in website materials. This model suggests that websites should foreshadow content even when it is not immediately accessible in order to engage users. It also points to particular usage patterns—brief initial visits followed by later visits of more duration [49]. The Transtheoretical/Stages of Change model may also hold promise in focusing the analysis of exposure and engagement. Velicer et al [51] suggest that users in action stage will access a program relatively more than users characterized as being in early stages (precontemplation, contemplation) or the later maintenance stage. Similarly, it might be helpful to consider the behavioral self-management model [17,52,53], which suggests that users who become more confident and capable in their self-management skills would tend to access a program less over time.

We view exposure as representing one of a set of complementary measures of the broader theme of program engagement. Other engagement measures include participant comprehension of program content, practice of that content (especially in the participant's everyday routines outside of interacting with the Web-based program), self-reported satisfaction with the function and content of the website, and measures of self-efficacy. While exposure is obviously important (indeed, it is best viewed as a prerequisite), it represents only one piece of the puzzle in seeking to understand program effectiveness.

## Appendix 1

**Table 4:** Table of detailed measures of program exposure

**Email prompts**	a) Percentage of participants receiving email prompts	Programmatic rules defined the timing of email prompts regarding completion of online assessments that were sent to participants in both conditions. Participants in the Enhanced condition also were sent "treatment-related" email prompts that contained tailored content related to quitting plan, quit date, and support of continued abstinence.
**Participant visits**	b) Number of visits	Visits by each participant to access smokeless tobacco cessation content on their assigned website were counted. All visits in which only online assessments were accessed were excluded.
	c) Aggregate duration of visits	Duration of each visit was defined as the sum of Web page durations during that visit. With one exception (noted next), Web page durations were defined as their logged end time minus start time. Because our program logic did not include a session-expiration feature that automatically logged out after a period of inactivity, we conservatively approximated the end of the visit by using the date/time of the last Web page that had been accessed before the abrupt end of the session. In addition, we followed the operational definition for visit expiration recommended by Peterson; that is, any Web page viewed for 30 minutes or more was defined as having ended the visit using the ending date/time stamp of the Web page that immediately preceded the hiatus. Moreover, if, after the hiatus, the participant resumed activity, it was considered to be a new visit for measurement purposes.
	d) Number of daily visits post-enrollment	The number of daily visits per participant was counted with "days" being defined in terms of their occurrence relative to the participant enrollment date. Participants could have more than one visit per day, and visits were defined using Peterson's recommendation (see above). Total visits per post-enrollment date aggregated these data across participants.
	e) Number of days of program access post-enrollment	The number of days of post-enrollment access to smokeless tobacco cessation content was defined for each participant as the last visit date minus the enrollment date.
	f) Plan to Quit	Participants in the Enhanced condition were given the opportunity to define a personal quitting plan and quit date. Although the program allowed participants to define their quitting plan and date multiple times, for purposes of the analyses in this report, we focused only on the first recorded date when a participant defined his/her quitting plan/date.
	g) Smokeless tobacco quit status	Participants in the Enhanced condition were able to indicate that they had quit using smokeless tobacco. This report enabled them to access "Staying Quit" content.
**Web page views**	h) Overall number of Web page views	The total number of Web pages viewed was logged.
	i) Specific page views (selected smokeless tobacco cessation content)	The total number of selected Web pages viewed related to smokeless tobacco cessation was logged, and a subset of these is described in this report.
	j) Web forum postings	Individual forum postings—in each of the two forums—were logged for each participant (Enhanced condition only).
